# Supplementary material for: Multidrug-Resistant Klebsiella pneumoniae Causing Severe Infections in the Neuro-ICU
Source: Antibiotics (Basel). 2021 Aug 13;10(8):979. doi: 10.3390/antibiotics10080979 (PMC8389041; doi:10.3390/antibiotics10080979)
Supplement: Supplementary file 1 [file antibiotics-10-00979-s001.zip › antibiotics-1321960-supplementary.pdf]

Table S1. Minimal inhibitory concentrations (MICs) of antimicrobials for *K. pneumoniae* clinical isolates.

Isolate	Patient	MICs of Antibacterials																				Phenotype
		AMP	SAM	CXM	FOX	CRO	CAZ	SCF	FEP	ETP	IPM	TET	TGC	CIP	CHL	GEN	TOB	AMK	SXT	NIT	CST	
B-548/18	D	>32	>32	>64	>64	>64	>64	>64	>64	>8	8	>16	>8	>4	>64	>64	>16	>64	>320	256	<0.5	XDR
B-784/18	D	>32	>32	>64	>64	>64	>64	>64	>64	>8	8	>16	>8	>4	>64	>16	>16	>64	>320	256	<0.5	XDR
B-1154/18	D	>32	>32	>64	>64	>64	>64	>64	>64	>8	4	>16	4	>4	>64	>16	>16	>64	>320	128	<0.5	XDR
B-1396/18-2	J	>32	>32	>64	>64	>64	>64	>64	>64	ND	8	>16	2	>4	>64	>16	>16	<2	>320	>512	<0.5	XDR
B-1618/18	Q	>32	>32	>64	8	>64	>64	>64	>64	4	<1	>16	4	>4	>64	<1	>16	<2	>320	128	<0.5	XDR
B-2035K/18-2	P	>32	>32	>64	>64	>64	>64	>64	>64	4	<1	>16	>8	>4	>64	>16	>16	>64	160	256	<0.5	XDR
B-2062/18	P	>32	16	>64	ND	ND	>64	>64	>64	ND	2	ND	ND	>4	>64	>16	>16	>64	160	256	<0.5	XDR
B-2086/18	P	>32	>32	>64	>64	>64	>64	>64	>64	4	<1	>16	>8	>4	>64	>16	>16	>64	160	256	<0.5	XDR
B-968/18-1	M	>32	>32	>64	>64	>64	>64	>64	>64	>8	<1	>16	4	>4	>64	<1	8	<2	80	256	<0.5	XDR
B-1120K/18	M	>32	>32	>64	>64	>64	>64	>64	>64	>8	<1	>16	4	>4	>64	<1	8	<2	80	256	<0.5	XDR
B-2625/18	S	>32	>32	>64	>64	ND	>64	>64	>64	>8	>16	>16	>8	>4	>64	>16	>16	<2	<20	128	<0.5	XDR
B-14/19	S	>32	>32	>64	>64	ND	>64	>64	32	>8	>16	>16	>8	>4	>64	>16	>16	<2	<20	128	<0.5	XDR
B-21/19	S	>32	>32	>64	>64	ND	>64	>64	32	>8	>16	>16	>8	>4	>64	>16	>16	<2	<20	128	<0.5	XDR
B-1398/18-1	L	>32	16	>64	<4	>64	16	<8	2	<0.5	<1	>16	1	<0.5	>64	<1	<1	<2	>320	64	<0.5	XDR
B-1406/18-1	L	>32	16	>64	<4	>64	16	<8	2	<0.5	<1	>16	1	<0.5	>64	<1	<1	<2	>320	64	<0.5	XDR
B-1412/18-1	L	>32	16	>64	<4	>64	8	<8	2	<0.5	<1	>16	1	<0.5	>64	<1	<1	<2	>320	128	<0.5	XDR
B-1230/18-1	K	>32	4	2	<4	<1	<1	<8	<1	<0.5	<1	<0.5	<0.5	<0.25	32	<1	<1	<2	<20	256	<0.5	R
B-849/18-2	K	16	4	2	<4	<1	<1	<8	<1	<0.5	<1	<0.5	<0.5	<0.25	>64	<1	<1	<2	<20	128	<0.5	R
B-1207/18	N	>32	8	>64	<4	32	4	<8	<1	<0.5	<1	<0.5	<0.5	<0.25	4	<1	<1	<2	<20	32	<0.5	R
B-1636/18	R	>32	4	2	<4	<1	<1	<8	<1	<0.5	<1	<0.5	<0.5	<0.25	4	<1	<1	<2	<20	64	<0.5	R
B-2523/18	R	>32	4	2	<4	<1	<1	<8	<1	<0.5	<1	<0.5	<0.5	<0.25	4	<1	<1	<2	<20	32	<0.5	R
B-3002K/17	A	>32	>32	>64	>64	>64	>64	>64	>64	>8	<1	>16	>8	>4	>64	<1	>16	8	>320	>512	<0.5	XDR
B-3060K/17	A	>32	>32	>64	>64	>64	>64	>64	>64	>8	<1	>16	>8	>4	>64	<1	>16	8	>320	>512	<0.5	XDR
B-2016K/17	A	>32	>32	>64	>64	>64	>64	>64	>64	>8	<1	>16	>8	>4	>64	<1	>16	8	>320	>512	<0.5	XDR
B-3299/17	A	>32	>32	>64	>64	>64	>64	>64	>64	>8	<1	>16	>8	>4	>64	<1	>16	8	>320	>512	<0.5	XDR
B-1040/18-1	I	>32	>32	16	<4	<1	<1	<8	<1	2	<1	2	1	>4	>64	>16	>16	<2	80	256	<0.5	XDR
B-792/18	I	>32	>32	16	16	<1	<1	16	<1	4	2	2	1	>4	32	<1	<1	<2	<20	>512	<0.5	XDR
B-853/18-1	I	>32	>32	16	>64	<1	<1	32	<1	>8	>16	<0.5	<0.5	>4	16	<1	<1	<2	<20	>512	<0.5	XDR
B-1214/18-2	F	>32	>32	>64	<4	>64	>64	16	32	<0.5	<1	>16	2	>4	4	<1	8	<2	>320	64	<0.5	MDR
B-789/18-1	E	>32	>32	>64	>64	>64	>64	>64	>64	>8	<1	>16	1	>4	16	>64	>16	8	>320	256	<0.5	XDR
B-851/18-1	E	>32	>32	>64	>64	>64	>64	>64	>64	>8	<1	>16	1	>4	16	>64	>16	16	>320	256	<0.5	XDR
B-790/18-1	G	>32	>32	16	>64	<1	<1	32	<1	>8	>16	2	1	>4	>64	>16	>16	<2	80	256	<0.5	XDR
B-823/18-1	G	>32	>32	16	>64	<1	<1	16	<1	>8	>16	2	<0.5	>4	>64	>16	>16	<2	80	256	<0.5	XDR
B-702/18	C	>32	>32	>64	>64	>64	>64	>64	>64	ND	8	>16	>8	>4	>64	<1	>16	ND	>320	>512	<0.5	XDR
B-771/18	B	>32	>32	16	32	<1	<1	32	<1	>8	>16	2	2	>4	>64	>16	>16	<2	80	>512	<0.5	XDR
B-102/19	T	>32	>32	>64	>64	ND	<1	>64	2	>8	8	>16	>8	>4	>64	>16	>16	>64	>320	>512	<0.5	XDR

B-543/18	H	>32	>32	>64	>64	<1	<1	32	<1	>8	>16	4	1	>4	>64	>16	>16	<2	40	>512	<0.5	XDR
B-587/18	H	>32	>32	16	>64	<1	<1	32	<1	>8	>16	4	1	>4	>64	>16	>16	<2	80	>512	<0.5	XDR
B-775/18-1	H	>32	>32	4	<4	<1	<1	<8	<1	4	<1	1	1	>4	>64	>16	>16	<2	80	>512	<0.5	XDR
B-691/18-4	H	>32	>32	16	>64	<1	<1	32	<1	>8	2	1	<0.5	>4	32	<1	<1	<2	<20	>512	<0.5	XDR
B-1363/18-1	O	>32	>32	>64	>64	>64	>64	>64	16	>8	>16	8	2	>4	8	<1	>16	16	<20	256	<0.5	XDR

Abbreviation: AMP, ampicillin; SAM, ampicillin-sulbactam; CXM, cefuroxime; FOX, cefoxitin; CRO, ceftriaxone; CAZ, ceftazidime; SCF, cefoperazone-sulbactam; FEP, cefepime; ETP, ertapenem; IPM, imipenem; TET, tetracycline; TGC, tigecycline; CIP, ciprofloxacin; CHL, chloramphenicol; GEN, gentamicin; TOB, tobramycin; AMK, amikacin; SXT, trimethoprim-sulfamethoxazole; NIT, nitrofurantoin; CST, colistin; R, resistance; MDR, multi drug-resistance; XDR, extensively drug-resistant; green color, sensitive; yellow color, intermediate; pink color, resistant; ND, no data.
